# EEG Connectivity Diversity Differences between Children with Autism and Typically Developing Children: A Comparative Study

**DOI:** 10.3390/bioengineering10091030

**Published:** 2023-09-01

**Authors:** Jiannan Kang, Hongxiang Xie, Wenqin Mao, Juanmei Wu, Xiaoli Li, Xinling Geng

**Affiliations:** 1 Child Rehabilitation Division, Ningbo Rehabilitation Hospital, Ningbo 315040, China; 2 State Key Laboratory of Cognitive Neuroscience and Learning, Beijing Normal University, Beijing 100875, China; 3 School of Biomedical Engineering, Capital Medical University, Beijing 100069, China

**Keywords:** autism spectrum disorder (ASD), EEG, phase lag entropy (PLE), connectivity diversity

## Abstract

Autism spectrum disorder (ASD) is a neurodevelopmental disorder characterized by deficits in social interaction and communication, and repetitive or stereotyped behaviors. Previous studies have reported altered brain connectivity in ASD children compared to typically developing children. In this study, we investigated the diversity of connectivity patterns between children with ASD and typically developing children using phase lag entropy (PLE), a measure of the variability of phase differences between two time series. We also developed a novel wavelet-based PLE method for the calculation of PLE at specific scales. Our findings indicated that the diversity of connectivity in ASD children was higher than that in typically developing children at Delta and Alpha frequency bands, both within brain regions and across hemispheric brain regions. These findings provide insight into the underlying neural mechanisms of ASD and suggest that PLE may be a useful tool for investigating brain connectivity in ASD.

## 1. Introduction

Autism spectrum disorder is a generalized brain dysfunction characterized by difficulties in social interaction and limited and stereotyped behaviors [1,2], with high rates of medical comorbidities, a spectrum of severities, and high rehabilitation costs [3,4]. Resting-state EEG provides a window into spontaneous local and long-range neuronal synchronization and has been investigated in many previous ASD studies, but results are inconsistent. Some previous studies reported that the overall connectivity was abnormal and emphasized local over-connectivity. More recent studies emphasize a subtle mixture of both sub- and hyper-connectivity, leading to no uniform conclusion [5,6,7].

The current techniques used to measure connectivity in neurophysiological signals did not adequately consider the temporal dynamics of synchronous modes [8]. Indeed, the popular phase synchronization method used coherence [9] or phase locking [10] to calculate connectivity, assuming that it was stable throughout the measurement period [11]. The phase synchronization value was obtained by averaging the phase differences within a period of a few seconds, thereby ignoring the temporal dynamics within that time frame [12].

In fact, neural communications are inherently transient and non-stationary [13]. Synchronization emerges and dissipates on a sub-second timescale, and fluctuating functional connectivity patterns have been observed in resting-state EEG [14] and fMRI data [15]. In this study, we introduced a novel measurement method called phase lag entropy (PLE) to quantify the diversity of temporal patterns in phase relationships [16]. PLE is a new phase synchronization estimation algorithm that can extract synchronization information between neural signals at a sub-second time scale while effectively avoiding mutual cancellation of synchronization information. PLE combines both the phase lag index [17] and Shannon entropy theory [18]. Thus, PLE reflects the coupling between signals as composed of fixed or varying connectivity patterns, incorporating temporal dynamics into phase synchronization estimation. This enhances the time sensitivity of synchronization indicators and better reflects the essential connectivity diversity of neural signals [19].

To date, no study has investigated the diversity of brain connectivity in children with ASD. In this study we proposed the wavelet PLE (wPLE) to further explore abnormalities in brain connectivity diversity at specific scales in children with ASD. The findings of this study may provide insight into the underlying neural mechanisms of ASD and PLE may be a useful tool for investigating and characterizing brain connectivity abnormalities in individuals with ASD.

## 2. Materials and Methods

### 2.1. Participants

A total of 179 children aged 3−10 years were incorporated into the study including typically developing (TD) children (76 males and 14 females; mean ± SD age: 5.09 ± 2.27 years) and low-functional autistic children (71 males and 18 females; mean ± SD age: 5.17 ± 1.89 years) with no statistical differences in age or gender. TD children were recruited from a kindergarten. They did not have any mental disorders, nor any nervous system disease or serious physical disorder. ASD children were diagnosed by experienced psychiatrists using the psychoeducational profile (Third Edition) [20] and Diagnostic and Statistical Manual of Mental Disorders-5 criteria [21]. They had no serious physical disease, severe brain trauma, or history of febrile seizures; children did not receive any psychiatric medications. This study followed the ethical principles for medical research under the Declaration of Helsinki and was approved by the ethics committee of Ningbo Rehabilitation Hospital.

### 2.2. Data Acquisition

EEGs were recorded in a shielded room and all children sat on comfortable chairs, wearing EEG caps with eyes open during EEG recording. During the data collection process, a parent and a specialist were typically present alongside the children to monitor their condition and ensure data quality. The specialist would provide gentle reminders to the children to minimize blinking. Uncontrollable eye movement artifacts were subsequently eliminated during the preprocessing stage to ensure the cleanliness of the data. EEGs were recorded for approximately 5−10 min. A 128-channel HydroCel Sensor Net System (Electrical Geodesics, Inc., Eugene, OR, USA) was used for the study. The electrode impedance was kept below 50 kΩ throughout EEG acquisition. The sampling frequency was 1000 Hz, and the reference electrode was Cz.

### 2.3. Data Preprocessing

Matlab R2016a and EEGlab V13.5.4b were used for offline data analysis. After down-sampling EEG data to 200 Hz, a 1−45 Hz band-pass filter was employed. An independent component analysis (ICA) algorithm [22] was employed to remove eye blink, muscular artifact, and electromyogram. All channels were re-referenced to an average reference, and 62 electrodes were used according to the 10–10 electrode system for the subsequent analysis.

Five brain lobes were selected to calculate their connectivity diversity within brain regions, including frontal lobe (F), left temporal lobe (LT), parietal lobe (P), right temporal lobe (RT), and occipital lobe (O). Eight brain lobes were selected to calculate across hemispheric connectivity diversity, including left frontal lobe (LF), right frontal lobe (RF), left temporal lobe (LT), right temporal lobe (RT), left parietal lobe (LP), right parietal lobe (RP), left occipital lobe (LO), and right occipital lobe (RO). Detailed electrode placement and connectivity settings is referred to [23].

A time window of 10 s (2000 sampling points) was selected to divide EEG signal into smaller epochs. For all samples, the number of epochs is around 6–10.

### 2.4. Phase Lag Entropy (PLE)

The phase lag entropy method combines the temporal dynamics of instantaneous phase with phase synchronization analysis. The calculation process of PLE algorithm is as follows [16]:

1. Extracting the phase difference ∆φ(t) of two EEG signals X(t) and $Y\left(t\right)$ through Hilbert transform;
2. Converting the phase difference ∆φ(t) into binary form $m_{t}$, which represents the symbolic result of the phase relationship between signals X(t) and Y(t) at time t. The symbolization rule is as follows: if $X\left(t\right)$ leads Y(t), i.e., $∆\varphi \left(t\right)>0$, then set the symbol parameter $m_{t}=1$; If $X\left(t\right)$ lags behind Y(t), i.e., $∆\varphi \left(t\right)<0$, then set the symbol parameter $m_{t}=0$.
3. Defining the temporal dynamic pattern of the phase relationship between signals $X\left(t\right)$ and Y(t):
  (1) $$M_{t}=\{m_{t},m_{t+\tau },m_{t+2\tau }\ldots \ldots m_{t+(l-1)\tau }\}$$
  where l and τ represent the embedding dimension and time lag parameters, respectively. For a specific l, the number of all possible patterns that may occur in the phase relationship is $2^{l}$. In this study, we choose *l* = 3 (dimensionless) and τ = 6 (unit: timepoints).
4. Based on the dynamic phase relationship pattern and the theory of Shannon entropy, the definition of PLE is as follows [16]:

(2) $$PLE=-\frac{1}{log(2^{l})}\sum_{i}p_{i}log\left(p_{i}\right)$$

where $p_{i}$ represents the probability of the i-th dynamic pattern of phase relationship and its value range is 0–1. If all possible patterns have equal probabilities, PLE tends to be 1. If some patterns dominate, then PLE tends towards 0. Higher PLE values indicate higher diversity or complexity of the temporal patterns in the phase relationships, while lower PLE values indicate more regular or predictable phase dynamics.

In this study, the preprocessed EEG epochs were further filtered into four different frequency bands, that is Delta (1–4 Hz), Theta (4–8 Hz), Alpha (8–13 Hz), and Beta (13–30 Hz). Then, the above steps were implemented to calculate PLE values at either within or across hemispheric brain regions. During subsequent statistical analysis, the PLE values of all epochs were further averaged.

### 2.5. Wavelet Phase Lag Entropy (wPLE)

In order to study the phase diversity at a more detailed frequency resolution, continuous wavelet transform was used to extract the phase of EEG signals at different frequency points, and then the phase lag entropy at each frequency point was calculated. The complex Morlet wavelet was used, and its definition formula is as follows [24]:

(3) $$\Psi (t)=\frac{1}{\sqrt{\pi B}}{exp}^{\frac{-t^{2}}{B}}{exp}^{j2\pi f_{c}t}$$

where *B* represents bandwidth and $f_{c}$ represents center frequency. *B* = 1 and *C* = 1.5 were selected in this study.

In wavelet analysis, the method of associating scale with frequency is to determine the center frequency *f_c_* of the wavelet [25]:

(4) fa=fca

where *a* represents the scale, *f_c_* represents the center frequency, and *f_a_* represents the pseudo frequency corresponding to scale *a*. From the above equation, it can be seen that the scale is inversely proportional to the pseudo frequency, with 1–30 Hz corresponding to a scale ranging from 300 to 10. If the scale increases, the wavelet will become more dispersed, resulting in lower pseudo frequencies.

Unlike the calculation of PLE, continuous wavelet transform [26] was conducted on each epoch of 62-channel EEG data at 30 scales, resulting in a wavelet coefficient matrix of 2000 × 62 × 30. At each scale, extract the phase difference ∆φ between the wavelet coefficients of each two channels. After symbolization ∆φ, the Shannon entropy is calculated to obtain the phase connectivity diversity at each pseudo frequency point from 1 to 30 Hz. This process was referred to as wPLE. The wPLE values were averaged among all epochs within brain region or across hemispheric brain region for subsequent analysis.

### 2.6. Statistical Analysis

For both within brain regions and across hemispheric regions, a repeated-measure analysis of variance (ANOVA) [27] was performed to compare the differences of PLE/wPLE values between TD and ASD groups at each frequency-band/scale. For significant group difference after ANOVAs, a post-hoc multiple comparison using the Tukey–Kramer test was conducted to further determine the group difference at specific brain region. Statistics were considered significant at *p* < 0.05. All statistical analyses were performed in Matlab.

## 3. Results

### 3.1. PLE Differences of Two Groups—Within Brain Regions

We first investigated the differences of connectivity diversity between TD and ASD children within the brain regions using PLE. Table 1 showed that there were significant differences in PLE between the TD group and the ASD group in the Theta and Alpha frequency bands. Additionally, significant differences in PLE were observed across different regions for all frequency bands. The interaction effect of group and brain region was significant in PLE specifically in the Alpha and Beta frequency bands.

**Figure 1:**
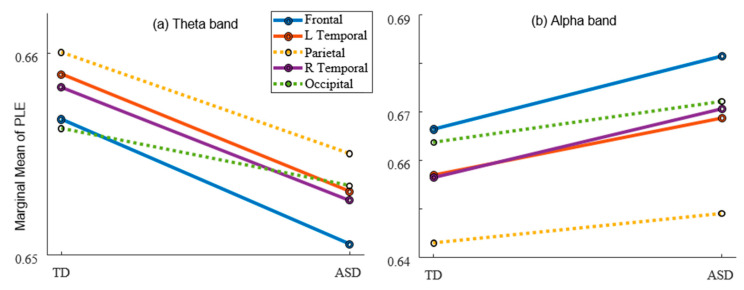
Post-hoc comparison of PLE between TD and ASD groups within brain regions in the (**a**) Theta band and (**b**) Alpha band. A solid line denotes a significant difference while a dotted line denotes an insignificant difference. Colors indicate different brain regions as depicted in the figure legend.

The post-hoc multiple comparison results of marginal means of PLE within each brain region were presented in Figure 1. In the Theta frequency band, compared to the TD group, the PLE in the frontal lobe (*p* = 0.002), left temporal lobe (*p* = 0.007), and right temporal lobe (*p* = 0.009) were significantly lower in the ASD group. Conversely, in the Alpha frequency band, the PLE value in these three brain regions were significantly higher in the ASD group (frontal: *p* = 0.000; left temporal: *p* = 0.003; right temporal: *p* = 0.000) compared to the TD group.

### 3.2. PLE Differences of Two Groups—Across Hemispheric Brain Regions

**Figure 2:**
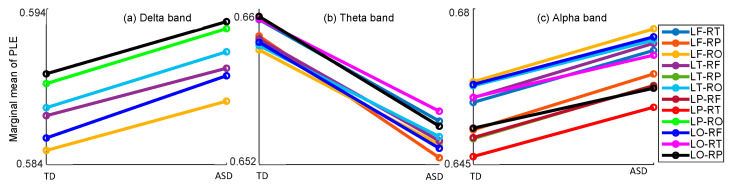
Post-hoc comparison of PLE between TD and ASD groups across hemispheric brain regions in the (**a**) Delta band, (**b**) Theta band, and (**c**) Alpha band. Only the regions with significant group differences are plotted with solid lines. Colors indicate different brain regions as depicted in the figure legend.

The connectivity diversity across hemispheric brain regions was further examined, and the results were presented in Table 2. Significant group differences in PLE were observed in the Delta, Theta, and Alpha frequency bands. The post-hoc comparison results, depicted in Figure 2, revealed that in the Delta frequency band, the PLE values were significantly higher in the ASD group compared to the TD group across six hemispheric brain regions (LF-RO: *p* = 0.041; LT-RF: *p* = 0.026; LT-RO: *p* = 0.007; LP-RO: *p* = 0.045; LO-RP: *p* = 0.030). Similarly, in Alpha frequency band, the PLE values were significantly higher in the ASD group compared to the TD group across eleven hemispheric brain regions (LF-RT: *p* = 0.004; LF-RP: *p* = 0.004; LF-RO: *p* = 0.002; LT-RF: *p* = 0.002; LT-RP: *p* = 0.005; LP-RF: *p* = 0.009; LP-RT: *p* = 0.014; LO-RF: *p* = 0.009; LO-RT: *p* = 0.011; LO-RP: *p* = 0.025). Conversely, in the Theta frequency band, the PLE values were significantly lower in the ASD group than that in the TD group across eight hemispheric brain regions (LF-RT: *p* = 0.015; LF-RP: *p* = 0.013; LF-RO: *p* = 0.027; LT-RF: *p* = 0.018; LT-RO: *p* = 0.028; LO-RF: *p* = 0.011; LO-RT: *p* = 0.029; LO-RP: *p* = 0.028).

### 3.3. wPLE Differences of Two Groups—Within Brain Regions

**Figure 3:**
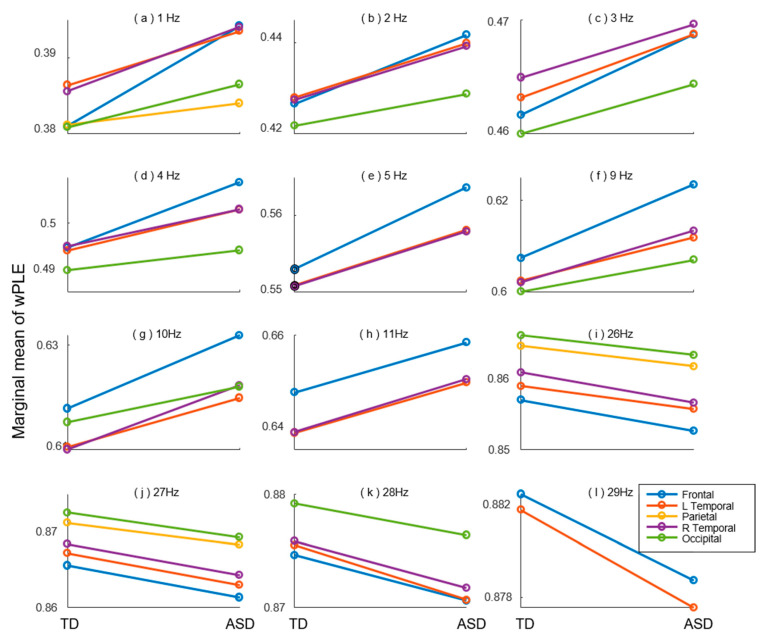
Post-hoc comparison of wPLE between TD and ASD groups within brain regions at (**a**) 1 Hz, (**b**) 2 Hz, (**c**) 3 Hz, (**d**) 4 Hz, (**e**) 5 Hz, (**f**) 9 Hz, (**g**) 10 Hz, (**h**) 11 Hz, (**i**) 26 Hz, (**j**) 27 Hz, (**k**) 28 Hz, (**l**) 29 Hz. Only the regions with significant group differences are plotted with solid line. Colors indicate different brain regions as depicted in figure legend.

We conducted further investigations to examine the differences of connectivity diversity between TD and ASD children within the brain regions using wPLE. The results presented in Table 3 revealed significant group differences at 12 scales, corresponding to pseudo frequency range of 1–5 Hz, 9–11 Hz, and 26–29 Hz. Figure 3 illustrated the post-hoc comparison results at these specific scales.

Compared to TD group, the wPLE values in ASD group exhibited a significantly increase in the pseudo frequency range of 1–5 Hz and 9–11 Hz, primarily in the frontal lobe, left temporal lobe, and right temporal lobe. Additionally, there were increases observed in the parietal and occipital lobes at several scales. Conversely, in the pseudo frequency range of 26–29 Hz, the wPLE value of ASD children were significantly decreased compared to TD children across almost all five brain regions.

### 3.4. wPLE Differences of Two Groups—Across Hemispheric Brain Regions

**Figure 4:**
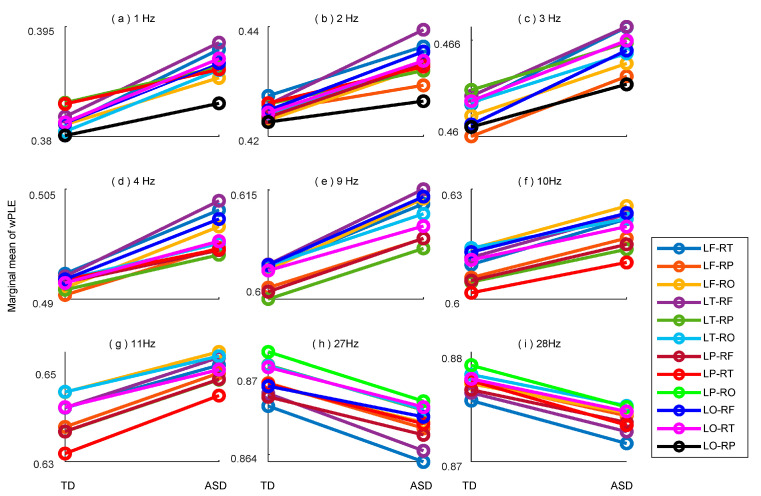
Post-hoc comparison of wPLE between TD and ASD groups across hemispheric brain regions at (**a**) 1 Hz, (**b**) 2 Hz, (**c**) 3 Hz, (**d**) 4 Hz, (**e**) 9 Hz, (**f**) 10 Hz, (**g**) 11 Hz, (**h**) 27 Hz, (**i**) 28 Hz. Only the regions with significant group differences are plotted with solid line. Colors indicate different brain regions as depicted in the figure legend.

Finally, we investigated the connectivity diversity across hemispheric brain regions using wPLE. The results displayed in Table 4 indicated significant group differences at nine scales, corresponding to the pseudo frequency range of 1–4 Hz, 9–11 Hz, and 26–28 Hz. The post-hoc comparison results in Figure 4 demonstrated that the wPLE values across most hemispheric brain regions exhibited a similar tendency to the within brain regions.

Specifically, in the pseudo frequency range of 1–4 Hz and 9–11 Hz, the wPLE values in ASD group were significantly higher compared to the TD group. Conversely, in the pseudo frequency range of 26–29 Hz, the wPLE values in the ASD group were significantly lower than those in the TD group.

## 4. Discussion

Phase lag entropy is a method used to quantify the complexity or irregularity of two time series based on their phase difference. Recently, it has been utilized to analyze anesthetized brain signals [28,29]. To our knowledge, there have been no studies using this method to calculate the differences between two groups. In this study, we used the PLE method to calculate differences in brain connectivity diversity between children with autism and TD children. Compared to typically developing children, children with autism showed a significant increase in connectivity changes at Delta and Alpha frequency bands, and a significant decrease in Theta bands. These findings provide insight into the underlying neural mechanisms of ASD and suggest that PLE may be a useful tool for investigating the abnormal brain connectivity in ASD.

Furthermore, the present study proposed an improved version of phase lag entropy that utilized wavelet analysis to determine the diversity of the phase relationship. This approach overcomes the limitations of conventional fixed frequency band division, which often smears frequency information during the filtering process. By employing continuous wavelet transform, this method can extract phase differences of the signals at multiple scales through expansion and translation operations. Consequently, the wavelet phase lag entropy (wPLE) method could identify subtle differences in connectivity diversity between two groups of children. Our findings indicated that the connectivity diversity of autistic children was higher than that of TD children within the pseudo frequency ranges of 1–4 Hz and 9–12 Hz, which correspond to canonical Delta and Alpha frequency bands, respectively. These results are consistent with the findings of the PLE analysis. Furthermore, the results of wPLE also indicate that children with ASD had lower brain connectivity diversity compared to TD children in the pseudo frequency range of 26–29 Hz, which belongs to the Beta frequency band. This difference was not evident in the results of PLE, reflecting, suggesting that wPLE may be capable of detecting subtle differences in brain connectivity diversity that PLE cannot capture.

This study suggested PLE and wavelet-based PLE may be useful tools to characterize the differences of connectivity diversity between ASD and TD groups. However, there are some limitations to consider. First, some autistic children did not complete the usual confirmatory instruments, such as the ADOS. As a result, our subjects might include those with many genetic disorders, which can affect the results. Second, this connectivity analysis was conducted in electrode-based brain regions, and a source localization tool was not used. This limitation may impact the accuracy and specificity of the findings. Third, our results are applicable only to restricted age range, and the lack of EEG data of infants and adolescents prevents generalizations across different age groups. Finally, this study only used the continuous complex Morlet wavelet transform to extract phase lag information. In future studies, it would be beneficial to explore the application of alternative wavelet functions in order to conduct a more comprehensive investigation of the phase lag entropy method and expand its application areas.

## Figures and Tables

**Table 1:** Results of repeated measurement ANOVA for PLE (within brain regions).

Freq. Band	Group (d = 1)			Region (d = 4)			Group * Region (d = 4)		
	F	*p*	$\eta_{p}^{2}$	F	*p*	$\eta_{p}^{2}$	F	*p*	$\eta_{p}^{2}$
Delta	2.753	0.099	0.015	43.592	**0.000**	0.198	1.322	0.260	0.007
Theta	6.270	**0.013**	0.034	8.492	**0.000**	0.046	1.800	0.127	0.010
Alpha	8.601	**0.004**	0.046	143.646	**0.000**	0.448	4.800	**0.001**	0.026
Beta	0.546	0.461	0.003	6.292	**0.000**	0.034	3.954	**0.004**	0.022

**Table 2:** Results of repeated measurement ANOVA for PLE (across hemispheric brain regions).

Freq. Band	Group (d = 1)			Region (d = 11)			Group * Region (d = 11)		
	F	*p*	$\eta_{p}^{2}$	F	*p*	$\eta_{p}^{2}$	F	*p*	$\eta_{p}^{2}$
Delta	4.532	**0.035**	0.025	15.130	**0.000**	0.079	1.078	0.375	0.006
Theta	4.907	**0.028**	0.027	8.400	**0.000**	0.045	1.021	0.425	0.006
Alpha	7.536	**0.007**	0.041	67.335	**0.000**	0.276	1.826	**0.045**	0.010
Beta	0.006	0.937	0.000	8.748	**0.000**	0.047	2.727	**0.002**	0.015

**Table 3:** Results of repeated measurement ANOVA for wPLE (within brain regions).

Pseudo Frequency (Hz)	Group (d = 1)			Region (d = 4)			Group * Region (d = 4)		
	F	*p*	$\eta_{p}^{2}$	F	*p*	$\eta_{p}^{2}$	F	*p*	$\eta_{p}^{2}$
1	22.133	**0.000**	0.111	22.236	**0.000**	0.112	6.807	**0.000**	0.037
2	38.654	**0.000**	0.179	51.426	**0.000**	0.225	13.871	**0.000**	0.073
3	23.497	**0.000**	0.117	24.507	**0.000**	0.122	5.007	**0.001**	0.028
4	30.015	**0.000**	0.145	61.209	**0.000**	0.257	12.779	**0.000**	0.067
5	7.392	**0.007**	0.040	36.189	**0.000**	0.170	9.454	**0.000**	0.051
6	0.244	0.622	0.001	13.620	**0.000**	0.071	0.799	0.526	0.004
7	1.194	0.276	0.007	14.655	**0.000**	0.076	2.915	**0.021**	0.016
8	1.180	0.279	0.007	9.827	**0.000**	0.053	1.298	0.269	0.007
9	7.261	**0.008**	0.039	94.864	**0.000**	0.349	10.589	**0.000**	0.056
10	8.878	**0.003**	0.048	111.539	**0.000**	0.387	6.910	**0.000**	0.038
11	5.626	**0.019**	0.031	61.102	**0.000**	0.257	1.977	0.096	0.011
12	3.557	0.061	0.020	14.175	**0.000**	0.074	1.668	0.156	0.009
13	0.131	0.718	0.001	36.540	**0.000**	0.171	2.019	0.090	0.011
14	3.182	0.076	0.018	84.386	**0.000**	0.323	0.075	0.990	0.000
15	2.935	0.088	0.016	68.023	**0.000**	0.278	1.244	0.291	0.007
16	3.199	0.075	0.018	30.041	**0.000**	0.145	2.217	0.066	0.012
17	2.784	0.097	0.015	2.549	**0.038**	0.014	1.755	0.136	0.010
18	1.061	0.304	0.006	12.180	**0.000**	0.064	1.843	0.119	0.010
19	0.071	0.790	0.000	27.238	**0.000**	0.133	1.421	0.225	0.008
20	0.734	0.393	0.004	32.673	**0.000**	0.156	1.014	0.399	0.006
21	0.095	0.758	0.001	24.994	**0.000**	0.124	1.224	0.299	0.007
22	0.133	0.716	0.001	47.350	**0.000**	0.211	1.542	0.188	0.009
23	0.700	0.404	0.004	88.531	**0.000**	0.333	1.054	0.379	0.006
24	1.797	0.182	0.010	112.302	**0.000**	0.388	1.104	0.353	0.006
25	3.738	0.055	0.021	113.467	**0.000**	0.391	1.331	0.257	0.007
26	8.145	**0.005**	0.044	103.293	**0.000**	0.369	0.862	0.486	0.005
27	10.579	**0.001**	0.056	66.544	**0.000**	0.273	0.634	0.638	0.004
28	8.471	**0.004**	0.046	36.893	**0.000**	0.172	1.745	0.138	0.010
29	4.071	**0.045**	0.022	18.418	**0.000**	0.094	2.095	0.080	0.012
30	1.317	0.253	0.007	10.690	**0.000**	0.057	2.063	0.084	0.012

**Table 4:** Results of repeated measurement ANOVA for wPLE (across hemispheric brain regions).

Pseudo Frequency (Hz)	Group (d = 1)			Region (d = 11)			Group * Region (d = 11)		
	F	*p*	$\eta_{p}^{2}$	F	*p*	$\eta_{p}^{2}$	F	*p*	$\eta_{p}^{2}$
1	17.381	**0.000**	0.089	4.647	**0.000**	0.026	1.961	**0.029**	0.011
2	30.306	**0.000**	0.146	10.723	**0.000**	0.057	5.401	**0.000**	0.030
3	11.618	**0.001**	0.062	4.431	**0.000**	0.024	1.235	0.257	0.007
4	18.640	**0.000**	0.095	13.317	**0.000**	0.070	5.558	**0.000**	0.030
5	3.540	0.062	0.020	7.230	**0.000**	0.039	1.747	0.058	0.010
6	0.042	0.839	0.000	4.060	**0.000**	0.022	0.688	0.751	0.004
7	2.036	0.155	0.011	8.366	**0.000**	0.045	2.169	**0.014**	0.012
8	0.672	0.414	0.004	1.217	0.270	0.007	0.505	0.901	0.003
9	5.278	**0.023**	0.029	22.744	**0.000**	0.114	3.660	**0.000**	0.020
10	6.767	**0.010**	0.037	43.876	**0.000**	0.199	3.878	**0.000**	0.021
11	4.989	**0.027**	0.027	30.753	**0.000**	0.148	2.534	**0.004**	0.014
12	3.637	0.058	0.020	3.335	**0.000**	0.018	2.543	**0.003**	0.014
13	0.244	0.622	0.001	14.618	**0.000**	0.076	1.890	**0.036**	0.011
14	2.700	0.102	0.015	48.993	**0.000**	0.217	0.631	0.803	0.004
15	2.081	0.151	0.012	36.530	**0.000**	0.171	0.807	0.633	0.005
16	1.915	0.168	0.011	15.044	**0.000**	0.078	1.047	0.401	0.006
17	1.924	0.167	0.011	0.951	0.490	0.005	1.577	0.099	0.009
18	0.254	0.615	0.001	6.181	**0.000**	0.034	2.620	**0.003**	0.015
19	0.775	0.380	0.004	12.263	**0.000**	0.065	1.665	0.076	0.009
20	2.265	0.134	0.013	17.962	**0.000**	0.092	1.116	0.344	0.006
21	0.714	0.399	0.004	11.523	**0.000**	0.061	0.823	0.617	0.005
22	0.001	0.971	0.000	13.962	**0.000**	0.073	1.056	0.394	0.006
23	0.221	0.639	0.001	27.771	**0.000**	0.136	0.646	0.790	0.004
24	0.288	0.592	0.002	29.447	**0.000**	0.143	2.094	**0.018**	0.012
25	1.597	0.208	0.009	32.582	**0.000**	0.155	1.875	**0.038**	0.010
26	3.721	0.055	0.021	25.766	**0.000**	0.127	1.024	0.422	0.006
27	7.544	**0.007**	0.041	16.772	**0.000**	0.087	0.805	0.635	0.005
28	6.118	**0.014**	0.033	8.740	**0.000**	0.047	0.668	0.770	0.004
29	2.876	0.092	0.016	3.775	**0.000**	0.021	0.711	0.729	0.004
30	0.870	0.352	0.005	2.552	**0.003**	0.014	0.848	0.592	0.005

## Data Availability

The datasets generated and analyzed in this study are available from the corresponding author upon reasonable request.
